# Development of a Rapid and Simple Method for Preparing Tea-Leaf Saponins and Investigation on Their Surface Tension Differences Compared with Tea-Seed Saponins

**DOI:** 10.3390/molecules23071796

**Published:** 2018-07-20

**Authors:** Xiao-Lan Yu, Yong He

**Affiliations:** College of Biosystems Engineering and Food Science, Zhejiang University, Hangzhou 310058, China; xiao-lanyu@zju.edu.cn

**Keywords:** tea-leaf saponins, ultrasonic-assisted water extraction, acetone precipitation, surface tension, soil remediation, tea-seed saponins

## Abstract

The relative overcapacity in China's tea-leaf production and the potential application of tea-leaf saponins in soil remediation encouraged in-depth developments and comprehensive utilizations of tea-leaf resources. Through variables optimizations using Box–Behnken designs for ultrasonic power, temperature as well as ultrasonic treatment time in ultrasonic-assisted water extraction and single-variable experiments for acetone-extraction solution ratio in acetone precipitation, a rapid and simple method was developed for preparing tea-leaf saponins. Tea-leaf saponins with the concentration of 3.832 ± 0.055 mg/mL and the purity of 76.5% ± 1.13% were acquired under the optimal values of 78 w, 60 °C, 20 min and 0.1 ratio of acetone-extraction solution. Both Fourier transform-infrared (FT-IR) spectra and ultraviolet (UV) spectra revealed slight composition differences between tea-leaf saponins and tea-seed saponins, while these differences were not reflected in the critical micelle concentration (CMC) and the surface tension of tea-leaf saponins and tea-seed saponins, indicating there was no need to distinguish them at the CMC. Further research attention on where tea-leaf saponins were in low concentrations is deserved to discover whether they had differences in comparison with tea-seed saponins, which was beneficial to apply them in the phytoremediation of contaminated soils.

## 1. Introduction

Tea saponins, a type of oleanane pentacyclic triterpene saponin mixtures, contained in stems, leaves, flowers and seeds of tea (*Camellia sinensis*) [1,2,3] as well as other plants of the genus *Camellia*, such as *Camellia oleifera* [4,5], *Camellia japonica* [6] and *Camellia chekiang-oleosa* Hu. [7], are classified into tea-leaf saponins (foliatheasaponins) [8,9] and tea-seed saponins (theasaponins) [10,11,12]. In addition, due to their distributions, research has proven that there are some differences between the compositions of tea-leaf saponins and tea-seed saponins [8,9,10,11,12,13]. Currently, seed meals of *C. oleifera*, the by-product from *Camellia* oil (tea-seed oil) extractions, are the principal source of tea saponins, specifically tea-seed saponins [4,5,14]. There is less research on the extraction of saponins from tea leaves, both the precipitation and the application, although researchers have summed up current literature on tea saponins (taking tea-seed saponins and tea-leaf saponins as a whole) from preparations to applications as natural surfactants [15]. Follow-up studies focused simply on tea-leaf saponin are required. Therefore, it is of great significance to develop a simple and rapid method for preparing tea saponins from tea leaves, not only expanding the source of tea saponins. This would contribute to the extensive use of tea saponins due to their beneficial effects on hemolysis; antibacterial, anti-inflammatory, and anti-oxidation activates; inhibition of alcohol absorption [16,17,18,19,20]; and soil remediation abilities [21,22,23,24,25]. In addition, it would alleviate the relative surplus of tea-leaf production in some countries, for example, China.

Water extraction [26,27], organic solvent extraction [4,28,29,30], mixed solvent extraction [31], microwave-assisted extraction [5,32,33], ultrasonic-assisted extraction [34,35,36], supercritical extraction [37], aqueous enzymatic [38,39,40] and fermentation [41] are methods utilized for extracting saponins from tea-seed saponins. Although alcohol extraction, especially ethanol extraction, appears to bear a higher yield in tea-seed extractions [15], due to the difference of extraction objects, for instance, seed meals of *C. oleifera* for tea-seed saponins and leaves of *C. sinensis* for tea-leaf saponins, water extraction might be a better choice for extracting tea-leaf saponins, as inferred and discussed by Yu. and He. [15,42]. To improve the performance of water extraction, ultrasound, which has a better assistant performance compared with microwave and light-wave [15], was applied as an auxiliary method in this research. As for precipitation, the easiest and most convenient refined purification method, acetone precipitation, was selected for refining crude tea saponins from aspects of experiments’ operability, instrument and reagent’s price as well as purities of tea saponins.

Once tea-leaf saponins were prepared with the help of ultrasonic-assisted water extraction and acetone precipitation, their Fourier transform-infrared spectra (FT-IR) and ultraviolet (UV) spectra were measured and differences between those of tea-seed saponins were compared. Meanwhile, the surface tension of both tea-leaf saponins and tea-seed saponins under a series of concentrations was determined. These measurements verified whether a difference of the surface tension exists between tea-leaf saponins and tea-seed saponins, which are from distinct sources and have sight differences in compositions, owing to the importance of the surface tension in tea saponins’ effects on soil remediation as natural non-ionic surfactants. The surface tension of tea-seed saponins has been measured [43,44,45,46,47], while data of tea-leaf saponins are lacking.

This research aimed at rapidly preparing tea-leaf saponins through ultrasonic-assisted water extraction and acetone precipitation, and then clarifying whether a difference of the surface tension between tea-leaf saponins and tea-seed saponins exists, which not only provides the necessary basis of tea-leaf saponins, but also complements existing understanding of tea saponins as well as benefits the application of tea saponins on soil remediation.

## 2. Results and Discussion

### 2.1. Optimizations of Ultrasonic-Assisted Water Extraction

**Figure 1:**
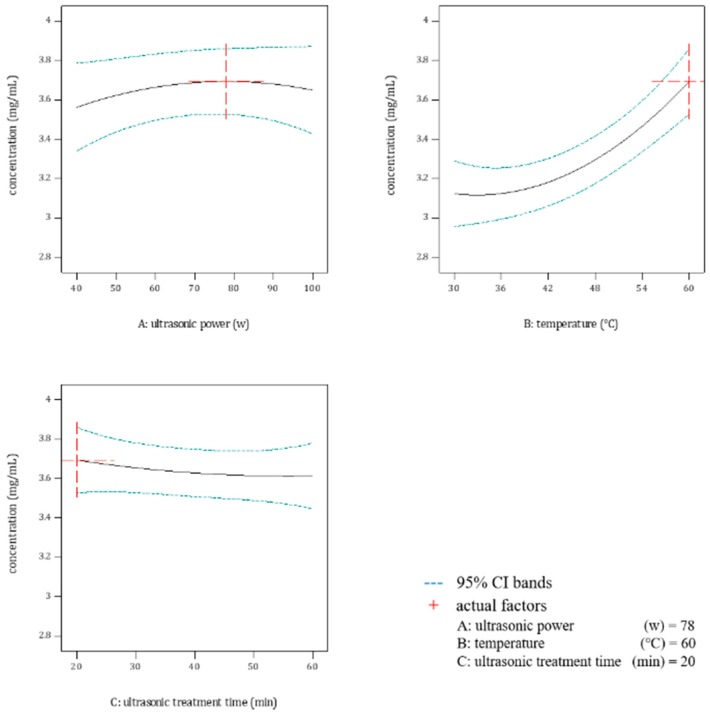
Effects of optimized variables on extracting tea-leaf saponins by Box–Behnken designs.

The concentration (*Y*) of each extraction solution is given in Table 1. Table 2 shows that variable A (ultrasonic power) and variable B (temperature) as well as the quadratic model was obviously significant; no interactions were found significant except variable B^2^, which just pointed out the effect of variable B (temperature), not the interaction between different variables. Because of the insignificance of variable C (ultrasonic treatment time), the minimum with the value of 20 min was selected as the optimum, considering factors of time saving, energy saving and effects of variable C (ultrasonic treatment time), as illustrated in Figure 1.

Effects of ultrasonic power, temperature and ultrasonic treatment time on the concentration of tea-leaf saponins in extraction solutions were not similar: quantitative effects of ultrasonic power and temperature belonged to quadratic curves while ultrasonic treatment time’s effect was linear. For quadratic curves, the optimal value of one variable had two choices: one was similar with linear effects, where one endpoint was the optimum; the other was different, i.e. the optimum fell between two endpoints. In this research, the optimum of ultrasonic power was the latter and the optimum of temperature was the former.

After analyzing and optimizing, the optimized extraction conditions for ultrasonic-assisted water extraction were acquired with 78 w for ultrasonic power, 60 °C for temperature and 20 min for ultrasonic treatment time. Under these optimized extraction conditions, the concentration of tea tree variety Jiukengzao in the extraction solution was 3.832 ± 0.055 mg/mL, having a 3.73% relative standard deviation (R.S.D) with the value predicted by Box–Behnken designs, which proved the predictive ability of the quadratic model provided by Box–Behnken designs.

Meanwhile, the concentration of 3.832 ± 0.055 mg/mL was not only higher than concentrations of all 15 runs in Box–Behnken designs, but also higher than the concentration obtained by the optimized water extraction using the same tea tree variety, whose value was 3.325 ± 0.044 mg/mL with a significant difference (*p* < 0.05), indicating ultrasound could indeed improve the extraction of tea-leaf saponins.

A 22.99% yield of tea-leaf saponins for tea tree variety Jiukengzao calculated from the concentration got under optimized extraction conditions (3.832 ± 0.055 mg/mL) was significantly higher (*p* < 0.05) than the yield of tea-seed saponins with the same method, ultrasonic-assisted water extraction based on optimizations, whose values was 21.32% [35], presenting that it was feasible to extract saponins from aged tea leaves, in favor of the utilization of excessive tea leaves, which could alleviate the relative surplus of tea-leaf production in China.

### 2.2. Optimizations of Acetone Precipitation

**Figure 2:**
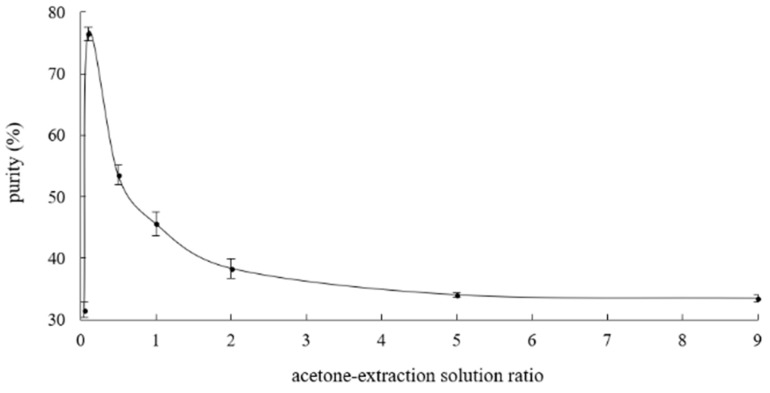
Effects of acetone-extraction solution ratio on purifying tea-leaf saponins by single-variable experiments.

For tea-leaf saponins solutions with the concentration equaled to 1 mg/mL, effects of acetone-extraction solution ratio on purifying tea-leaf saponins were investigated, as shown in Figure 2.

The acetone-extraction solution ratio ranged from 0.05 to 9; the tendency of its effect was ascending at first followed by a decline afterwards. The curve came to the peak at acetone-extraction solution ratio of 0.1. The optimal acetone-extraction solution ratio of 0.1 was affected by the concentration of the extraction solution and might change as it changed. Therefore, for extraction solutions whether of tea-leaf saponins or tea-seed saponins, in other concentrations, the optimal acetone-extraction solution ratio required a finer optimization.

Usually, existing research focused on the investigation of the ratio between acetone or other precipitants to extraction solutions without offering the data of extraction solutions’ concentrations [29,47,48], making it difficult to compare results. A broader range of extraction solution concentrations for acquiring the corresponding optimal values of ratio deserves more attention if it is beneficial to industrial purifications.

The purity of 76.5% ± 1.13% for tea-leaf saponins was probably not high enough, however, it was feasible to prepare high purity tea-leaf saponins solutions through calculations. It is worth noting that the purity of tea-seed saponins bought from Aladdin or Macklin is 10–25% and 20–40%, respectively, which not as high as we expected, suggesting the industrial purification of tea saponins, whether tea-leaf saponins or tea-seed saponins, needs optimizations.

### 2.3. Properties Differences Between Tea-Leaf Saponins and Tea-Seed Saponins

As kinds of saponins, both tea-leaf saponins and tea-seed saponins have common properties of saponins, such as hemolysis; fish toxicity, antibacterial, anti-inflammatory, and anti-oxidation activities; etc. [17,19,49,50,51,52,53,54]. Nevertheless, this study aimed at measuring the difference of tea-leaf saponins and tea-seed saponins on the surface tension, which contributes to the surface activity of tea saponins as natural surfactants beneficial for the remediation of contaminated soils. The potential difference on the surface tension possibly resulting from slight differences in the composition of tea-leaf saponins and tea-seed saponins was revealed by FT-IR spectra and UV spectra.

#### 2.3.1. Compositions Differences Reflected in FT-IR Spectra and UV Spectra

##### FT-IR Spectra

**Figure 3:**
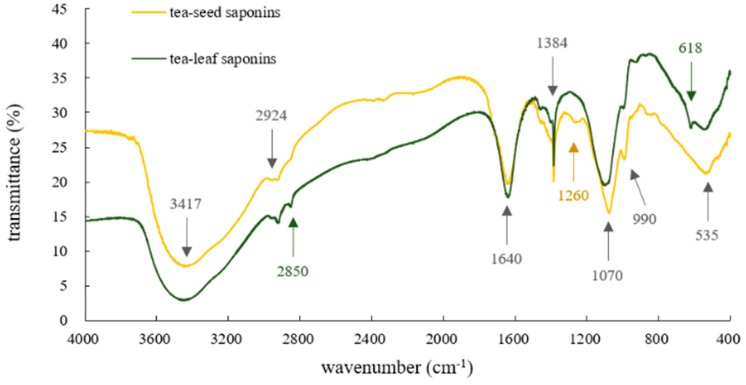
FT-IR spectra of tea-leaf saponins and tea-seed saponins. Wavenumbers are in yellow for tea-seed saponins, in green for tea-leaf saponins and in grey for both.

Figure 3 presents differences of tea-leaf saponins and tea-seed saponins in FT-IR spectra. Curves of tea-leaf saponins and tea-seed saponins were consistent in trend with local differences. The broad peak near 3417 cm^−1^ corresponded to the O–H stretching vibrations [44,55]; peaks of 2924 cm^−1^ and 2850 cm^−1^ were assigned to infrared CH_2_ symmetric stretching band [55,56]; and peaks 1640 cm^−1^, 1384 cm^−1^, 1260 cm^−1^, 1070 cm^−1^ and 535 cm^−1^ could be related to stretching vibration band of C=C in sapogenins, in-plane bending vibration of –OH, –C–O stretching vibrations of primary alcohols, and stretching vibration of C–O and C–Br [43,57,58], respectively. Peaks at 990 cm^−1^ and 618 cm^−1^ might be regarded as C–O bond stretching in the C–OH group and alkene mono-substitution, respectively, according to current literature [57]. In summary, it could be inferred that tea-leaf saponins had a stronger symmetric vibration of CH_n_, especially C–CH_2_ bonds, which indicated their composition differences in comparison with tea-seed saponins. The unique peak of tea-seed saponins at 1260 cm^−1^ suggested the existence of primary alcohols in tea-seed saponins.

##### UV Spectra

**Figure 4:**
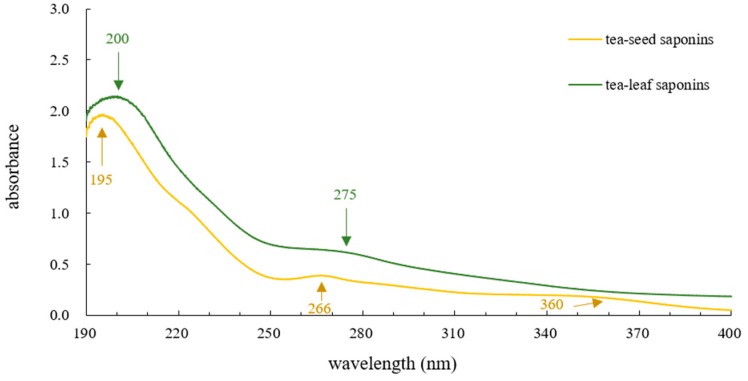
UV spectra of tea-leaf saponins and tea-seed saponins. Wavenumbers are in yellow for tea-seed saponins, and in green for tea-leaf saponins.

In UV spectra of tea-leaf saponins and tea-seed saponins, regardless of their differences in absorbances resulting from their distinct concentrations, the trends were consistent, as demonstrated in Figure 4. Compared with existing research [13,59,60], where the characteristic the highest peak of tea saponins was at 215 nm, peaks in this study showed shifts; for instance, the highest peak of tea-leaf saponins appeared at 200 nm while that for tea-seed saponins was 195 nm. Peaks at 266 nm and 360 nm of tea-seed saponins probably related to proteins, sugars or flavonoids in tea-seed saponins [59,60] due to their purities ranging from 10% to 25%. Nonetheless, the unique peak around 280 nm of tea-leaf saponins was regarded as the characteristic peak of tea-leaf saponins by researchers [13]. This peak was thought to be produced by the cinnamic acid [13], which only contained in tea-leaf saponins, and the strong absorption of tea saponins at 215 nm was generated from the α, β unsaturated conjugated double bonds at C-21 [60].

Both FT-IR spectra and UV spectra reflected composition differences between tea-leaf saponins and tea-seed saponins, but these differences were slight, and might not be enough for them to have different properties.

#### 2.3.2. Surface Tension Differences

**Figure 5:**
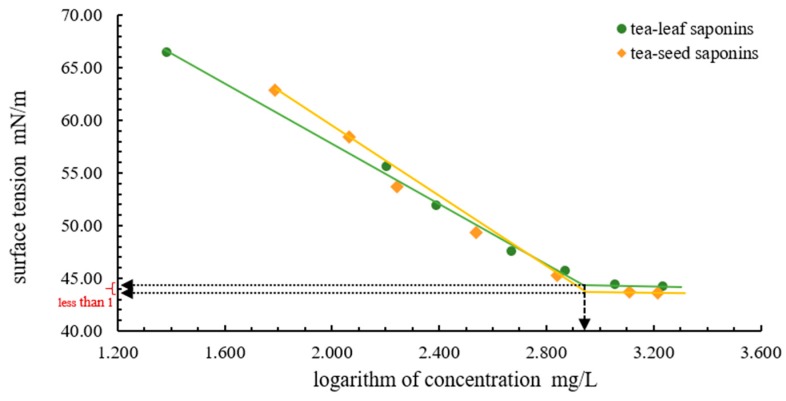
Surface tension versus concentration (γ–*C*) curves of tea-leaf saponins and tea-seed saponins.

The surface tension of tea-leaf saponins and tea-seed saponins showed differences (Figure 5); however, these differences were mild, even not statistically significant at several concentrations. In addition, tea-leaf saponins and tea-seed saponins enjoyed the same critical micelle concentration (CMC) with an approximate value of 880 mg/L (lg *C* = 2.945), and their corresponding surface tension’ differences were less than 1.

The same CMC as well as similar surface tension of tea-leaf saponins and tea-seed saponins indicated that there was no necessity to distinguish them when using them as natural surfactants at the CMC, whereas one point requires attention: according to current research, tea saponins have side effects on the growth of plant seeds and in vivo antioxidant system activities in plants when their concentrations exceed the safe concentration with an approximate value of 200 mg/L [61,62], which restricts concentrations of tea saponins utilized in soil remediation; meanwhile, the surface tension of tea-leaf saponins and tea-seed saponins below 200 mg/L (lg 200 = 2.301) seems to have some differences, making it necessary to measure whether there exist differences of tea-leaf saponins and tea-seed saponins on seeds growth or in vivo antioxidant system activities of hyperaccumulators under low concentrations, which contributed to the application of tea saponins on phytoremediation as they could enhance the bioavailability of heavy metals and improve the accumulation of heavy metals by hyperaccumulators.

Several methods were employed to measure the surface tension of tea saponins solutions and their results were distinct, as shown in Table 3. Even utilizing the same method, for example, Wilhelmy plate, results of the CMC and the surface tension were not the same [43,44], for which temperature of the experimental environment was perhaps the reason. Results of this study on the CMC and the surface tension of tea-leaf saponins and tea-seed saponins fell within results of the existing studies, suggesting their differences might come from the distinction of measurement methods along with the experimental environment, such as the temperature and the pH of tea saponins solutions.

## 3. Materials and Methods 

### 3.1. Materials

Tea tree (*Camellia sinensis*) variety Jiukengzao, whose leaf type, germination stage and adaptability are large, early and green tea [63], respectively, was chosen and its first leaves on the previous year’s twig (from top to bottom) were picked on 22 December 2017 as aged leaves from a pollution-free tea plantation (grant number: WNCR-ZJ16-12141) in Changxing County, Zhejiang Province, China. Then, tea leaves were wiped to remove dust, dried in an oven at 60 °C until they were in constant weights and sieved through a No. 60 mesh.

Bought from Sinopharm Chemical Reagent Co., Ltd, Shanghai, China, chemicals and reagents of analytical grade were utilized in this research. Deionized water (≥18.2 MΩ) was applied for the preparation of aqueous solutions.

Tea-seed saponins of biochemical reagent grade purchased from Macklin with purity between 10% and 25% served as the standard for comparisons with tea-leaf saponins prepared through the following procedures.

### 3.2. Box–Behnken Designs for Ultrasonic-Assisted Water Extraction

For ultrasonic-assisted water extraction, performance was compared not only before and after Box–Behnken designs, but also with water extraction optimized for tea-leaf saponins [42] whose extraction conditions were 75 mL/g liquid–solid ratio, 1 h extraction time and 80 °C extraction temperature. Ultrasonic power (w), temperature (°C) and ultrasonic treatment time (min) in ultrasonic-assisted water extraction were optimized in the three-level Box–Behnken designs with ranges designed consulting the maximum and minimum limits of the ultrasonic cleaner (SK2210HP, Shanghai Kudos Ultrasonic instrument Co., Ltd, Shanghai, China) (Table 1). The variety Jiukengzao is planted extensively in Zhejiang Province and possesses the highest yield of tea-leaf saponins among six tea tree varieties studied by Yu and He [42]; thus, Jiukengzao was selected in this research and 75 mL/g obtained from tea-leaf saponins water extraction based on optimizations [42] was set as the liquid–solid ratio. After filtering with a 0.45 μm microporous film, the concentration of each extraction solution (mg/mL), $c_{saponin}$, was determined with the aid of a double beam UV-visible spectrophotometer (TU-1901, Beijing Persee General Instrument Co., Ltd., Beijing, China) and calculated through the standard curve built by the well-established vanillin–sulfuric acid method [64]. $c_{saponin}$ was also employed as the dependent variable in Box–Behnken designs.

The significant level was 0.05 and data acquired from Box–Behnken designs were analyzed by Design-Expert 11 (Stat-Ease, Inc., Minneapolis, MN, USA).

### 3.3. Single-Variable Experiments for Acetone Precipitation

Effects of acetone-extraction solution ratio on the purity of tea-leaf saponins acquired from acetone precipitation was investigated through single-variable experiments, ranging from 0.05 to 9. The concentration of tea-leaf saponins used in acetone precipitation was 1 mg/mL. Each measurement was performed three times.

### 3.4. Measurements of FT-IR Spectra and UV Spectra

After extraction and purification, the powder of tea-leaf saponins was desiccated in the low-temperature vacuum drying (DZG-6050, Shanghai Sumsung Laboratory Instrument Co., Ltd., Shanghai, China) and stored in the desiccator.

FT-IR spectra and UV spectra of tea-leaf saponins and tea-seed saponins were recorded with a Nicolet™ iS™ 10 FT-IR spectrophotometer (Thermo Scientific, Waltham, MA, USA) using KBr pellets methods and a double beam UV-visible spectrophotometer (TU-1901, Beijing Persee General Instrument Co., Ltd., Beijing, China) using deionized water as the dissolvent. 

### 3.5. Measurements of the Surface Tension

Pendent drop method determined by a video optical contact angle surface and interface tension measuring instrument (A-100P, Maist Vision Inspection & Measurement Co., Ltd., Ningbo, China) was applied to measure the surface tension both of tea-leaf saponins and tea-seed saponins in a series of concentrations at 30 °C in aqueous medium, without the adjustment of pH. The volume of each drop was 5 μL.

CMC is the concentration point when an amphiphilic component in solution initiates to form micelles, being essential for biosurfactant applications, as concentrations of biosurfactants above the CMC, no further effect on the surface activities is expected [55]. In the plot of the surface tension (γ) in the unit of mN/m as a function of the logarithm of concentrations (mg/L) of tea-leaf saponins and tea-seed saponins, the CMC value was determined to be the intersection point between the two fitted straight lines of the pre-CMC and post-CMC data.

## 4. Conclusions 

A simple and rapid method for preparing tea-leaf saponins was developed. Ultrasonic-assisted water extraction and acetone precipitation based on optimizations performed well in extracting and purifying tea-leaf saponins with ultrasonic power, temperature as well as ultrasonic treatment time in ultrasonic-assisted water extraction optimized in Box–Behnken designs and acetone-extraction solution ratio in acetone precipitation optimized in single-variable experiments. Optimal values of 78 w, 60 °C, 20 min and 0.1 were chosen and then tea-leaf saponins with a higher concentration of 3.832 ± 0.055 mg/mL and a purity of 76.5% ± 1.13% were acquired. FT-IR spectra and UV spectra revealed composition differences between tea-leaf saponins and tea-seed saponins; however, they were not significant enough to produce statistical differences in the CMC, which was determined by the surface tension. The similarity of the CMC and the surface tension of tea-leaf saponins and tea-seed saponins indicated it is unnecessary to distinguish them at the CMC, while further research is required for tea-leaf saponins under low concentrations to figure out whether they are different from tea-seed saponins. Developing a simple and rapid method for preparing tea-leaf saponins and investigating their surface tension differences compared with tea-seed saponins not only verified the feasibility of extracting tea saponins from tea leaves, but also provided basic understandings of utilizing tea saponins in the application of phytoremediation for contaminated soils, which would contribute to alleviating the relative surplus of tea-leaf production in China and improving the utilization efficiency of tea-leaf resources.

## Figures and Tables

**Table 1:** Box–Behnken designs for tea-leaf saponins ultrasonic-assisted water extraction.

Run	A (w)	B (°C)	C (min)	*Y* (mg/mL)
**1**	70	45	40	3.216
**2**	40	45	60	3.212
**3**	70	30	20	3.081
**4**	100	45	20	3.156
**5**	70	60	20	3.704
**6**	100	60	40	3.513
**7**	40	30	40	3.055
**8**	40	45	20	3.190
**9**	100	45	60	2.837
**10**	100	30	40	2.890
**11**	70	60	60	3.708
**12**	70	45	40	3.160
**13**	70	30	60	2.935
**14**	70	45	40	3.089
**15**	40	60	40	3.543

A, ultrasonic power; B, temperature; C, ultrasonic treatment time; *Y*, extraction solution concentration.

**Table 2:** Analysis of Variance (ANOVA) for the quadratic model selected from Box–Behnken designs.

Source	Sum of Squares	d*f*	Mean Square	F-Value	*p*-Value
Model	1.04	9	0.1161	21.51	0.0018 **
A-ultrasonic Power	0.0456	1	1.04	8.45	0.0335 *
B-Temperature	0.7856	1	0.0456	145.58	< 0.0001 **
C-Ultrasonic Treatment Time	0.0241	1	0.7856	4.46	0.0883
AB	0.0046	1	0.0241	0.8843	0.4003
AC	0.0291	1	0.0046	5.39	0.0680
BC	0.0056	1	0.0291	1.04	0.3561
A^2^	0.0245	1	0.0056	4.54	0.0862
B^2^	0.1153	1	0.0245	21.37	0.0057 **
C^2^	0.0024	1	0.1153	0.4362	0.5382

* significant variable with *p* < 0.05; ** extremely significant variable with *p* < 0.01.

**Table 3:** Surface properties for tea saponins using different measurement methods.

Method	Temperature °C	CMC	γ (mN/m)	Reference
Wilhelmy plate ^1^	30 ± 5	pH = 6 0.11 g/L	37.6	[44]
		pH = 9 0.38 g/L	37.8	
		pH = 12 1.14 g/L	38.4	
Wilhelmy plate	20	0.63 g/L	36.99	[43]
Du Nouy ring method	20	1.814 g/L ^2^	43.5	[55]
maximum bubble pressure method	32 ± 0.2	0.5%	48.09	[47]
not mentioned	not mentioned	0.15%	30 ^3^	[45]
pendent drop method	30	0.88 g/L	43.80 ^4^ 44.54 ^4^	this study

^1^ also known as the hanging plate method; ^2^ calculated from the average molecular weight of 809.12 g/mol and the CMC of 2.242 mmol/L provided by the authors; ^3^ data read from the figure, not accurate; ^4^ 43.80 for tea-seed saponins and 44.54 for tea-leaf saponins.
